# Vinyl-Addition Homopolymeization of Norbornenes with Bromoalkyl Groups

**DOI:** 10.3390/polym15224444

**Published:** 2023-11-17

**Authors:** Artyom O. Lunin, Fedor A. Andreyanov, Igor S. Makarov, Maxim V. Bermeshev

**Affiliations:** A.V. Topchiev Institute of Petrochemical Synthesis, RAS, 29 Leninskiy Pr., 119991 Moscow, Russia; lunin@ips.ac.ru (A.O.L.); andreyanov@ips.ac.ru (F.A.A.); makarov@ips.ac.ru (I.S.M.)

**Keywords:** vinyl-addition polymerization, polynorbornene, norbornenes with bromoalkyl groups, Pd-catalysts, Pd-complexes with NHC-ligands

## Abstract

Vinyl-addition polynorbornenes are of great interest as versatile templates for the targeted design of polymer materials with desired properties. These polymers possess rigid and saturated backbones, which provide them with high thermal and chemical stability as well as high glass transition temperatures. Vinyl-addition polymers from norbornenes with bromoalkyl groups are widely used as precursors of anion exchange membranes; however, high-molecular-weight homopolymers from such monomers are often difficult to prepare. Herein, we report the systematic study of vinyl-addition polymerization of norbornenes with various bromoalkyl groups on Pd-catalysts bearing N-heterocyclic carbene ligands ((NHC)Pd-systems). Norbornenes with different lengths of hydrocarbon linker (one, two, and four CH_2_ groups) between the bicyclic norbornene moiety and the bromine atom were used as model monomers, while single- and three-component (NHC)Pd-systems were applied as catalysts. In vinyl-addition polymerization, the reactivity of the investigated monomers varied substantially. The relative reactivity of these monomers was assessed in copolymerization experiments, which showed that the closer the bromine is to the norbornene double-bond, the lower the monomer’s reactivity. The most reactive monomer was the norbornene derivative with the largest substituent (with the longest linker). Tuning the catalyst’s nature and the conditions of polymerization, we succeeded in synthesizing high-molecular-weight homopolymers from norbornenes with bromoalkyl groups (M_n_ up to 1.4 × 10^6^). The basic physico-chemical properties of the prepared polymers were studied and considered together with the results of vinyl-addition polymerization.

## 1. Introduction

**Figure 1:**
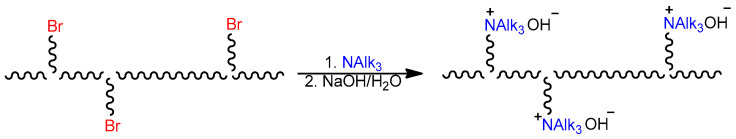
Modification of polymers bearing bromoalkyl groups with a tertiary amine.

Polymers bearing bromoalkyl groups in side chains are the versatile precursors of anion exchange membranes. Bromoalkyl groups in such polymers can be easily transformed into tetraalkylammonium functionalities via treatment with a tertiary amine (Figure 1). A key requirement for an anion exchange membrane is its stability in alkaline solutions, and the destruction of polymer backbones under such conditions may be one of the reasons for the poor stability of such a membrane. As a result, the right nature of a backbone is of significant importance. Polymers with saturated hydrocarbon backbones can meet this requirement. Several polymers with hydrocarbon main chains and containing bromoalkyl groups in side chains were characterized and tested as promising anion exchange membrane materials after modifications [1,2,3,4]. Such polymers showed improved stability under alkaline conditions combined with good ion conductivity. The rapid development of the industry has led to the emergence of another essential requirement: an anion exchange membrane must function at elevated temperatures, and the associated polymer should have, as a rule, a high glass temperature (T_g_ > 100 °C).

**Figure 2:**
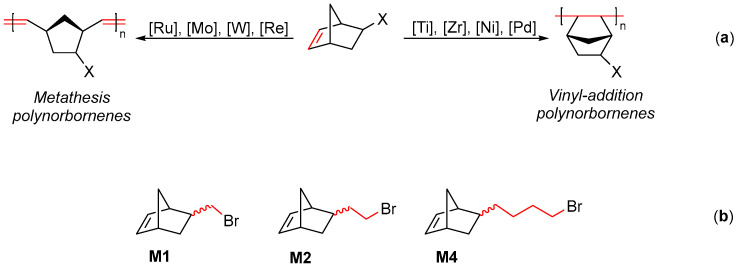
(**a**) The main methods of norbornenes’ polymerization and (**b**) the structure of the monomers used in this work (the number in the monomer abbreviation means the number of CH_2_-groups in the linker between the norbornene moiety and the bromine atom (e.g., in the case of M4, there are four CH_2_-groups in the linker).

Polynorbornenes with bromoalkyl groups in side chains are promising candidates for the preparation of anion exchange membranes [5,6,7,8] because of their rigid main chains, which provide high glass transition temperatures, and the hydrocarbon nature of the main chains (Figure 2a). In particular, vinyl-addition polynorbornenes have attracted a lot of attention as precursors of anion exchange membranes. These polymers contain saturated and more stiff main chains compared to metathesis isomers. In the literature, several vinyl-addition copolymers based on bromoalkyl-substituted norbornenes and anion exchange membranes derived from these polymers have been characterized [9,10]. The synthesis of these *co*polymers was mainly due to the moderate or low reactivity of bromoalkyl-substituted norbornenes in vinyl-addition polymerization, which leads to *homo*polymers with insufficient molecular weights to form robust free-standing thin films. To address this issue, a more reactive norbornene-type monomer is typically introduced during copolymerization of a bromoalkyl-substituted norbornene. On the one hand, the incorporation of a more reactive monomer allows molecular weights to increase, and, therefore, allows the mechanical properties of the resulting polymers to be enhanced. On the other hand, it is also possible to tune the hydrophilic/hydrophobic balance of a membrane, which becomes especially important after the modification of such a copolymer with a tertiary amine. As comonomers in the synthesis of copolymers based on bromoalkyl-substituted norbornene, norbornene and 5-alkyl-norbornene are usually utilized [7]. These monomers are inexpensive and extremely reactive. Nevertheless, the incorporation of these monomers reduces the density and concentration of ionogenic groups in a polymer material, while the use of homopolymers of bromoalkyl norbornenes will allow one to obtain an ionic group in each monomer unit. However, the synthesis of high-molecular-weight homopolymers of bromoalkyl norbornenes is difficult, limiting their utility as anion exchange membrane precursors. The most effective catalysts for vinyl-addition homopolymerization of bromoalkyl norbornenes produced the corresponding polymers with an M_n_ of no more than 1.2 × 10^5^ in the case of the monomer with four methylene groups in the bridge between norbornene and bromine (M4, Figure 2b), while for the related monomer with one methylene group in the bridge between norbornene and bromine (M1, Figure 2b), values of M_n_ were below 10^4^ [11,12].

Here, we report the detailed study of vinyl-addition homopolymerization of bromoalkyl norbornenes (M1–M4, Figure 2b) in the presence of Pd-catalysts bearing N-heterocyclic carbene ligands. An assessment of the relative monomers’ reactivity was performed. The tuning of catalyst’s nature and conditions of polymerizations made it possible to obtain high-molecular-weight homopolymers (e.g., with M_n_ > 10^6^ in the case of M4, Figure 2b) showing good film-forming properties. Such synthesized homopolymers of bromoalkyl norbornenes can become attractive precursors of new anion exchange membranes with enhanced anion conductivity. The properties of the polynorbornenes prepared were studied via DSC, WAXD and TGA analysis, and free volume distribution was evaluated by nitrogen low-temperature sorption and fractional free volume.

## 2. Materials and Methods

A description of the materials and the equipment used in this study is provided in Supplementary Material.

### 2.1. Synthesis of Monomers

#### 2.1.1. Synthesis of 5-(Bromomethyl)-2-norbornene (M1) [11]

Dicyclopentadiene (20.0 g, 151.5 mmol, 1 eq.), allyl bromide (73.3 g, 606 mmol, 4 eq.), and hydroquinone (6.0 mmol, 0.04 eq.) were added to an argonated ampoule. The ampoule was sealed and then heated at 170 °C for 7 h. The product was purified via successive distillations (10 Torr, 91–93 °C) to give 44.0 g of the desired product (yield: 80%).

^1^H NMR (CDCl_3_; δ, ppm): 6.27–5.94 (m, 2H), 3.49–3.16 (m, 1H), 3.11–2.94 (m, 2H), 2.92–2.83 (m, 2H), 2.00–1.81 (m, 1H), 1.52–1.17 (m, 3H), 0.66–0.51 (m, 1H).

^13^C NMR (CDCl_3_; δ, ppm): *endo*-isomer: 138.34, 131.72, 49.80, 45.61, 43.24, 42.23, 38.40, 32.91; *exo*-isomer: 137.13, 136.48, 45.96, 45.06, 42.53, 42.21, 39.34, 33.46.

MS (EI) [C_8_H_11_Br]: 186, 188 g/mol (calculated mass 186.08, 188.08 g/mol).

#### 2.1.2. Synthesis of 5-(2-Bromoethyl)-2-norbornene (M2) [11]

Cyclopentadiene (30.7 g, 464.8 mmol, 1 eq.), 4-bromobutene-1 (125.5 g, 929.5 mmol, 2 eq.), and hydroquinone (9.3 mmol, 0.02 eq.) were added to an argonated ampoule. The ampoule was sealed and then heated at 170 °C for 9 h. The product was purified via successive distillations (12 Torr, 103–105 °C) to give 35.0 g of the monomer (yield: 40%).

^1^H NMR (CDCl_3_; δ, ppm): 6.19–5.87 (m, 2H), 3.53–3.30 (m, 2H), 2.87–2.74 (m, 2H), 2.25–2.11 (m, 2H), 2.05–1.77 (m, 1H), 1.77–1.19 (m, 5H), 0.59–0.47 (m, 1H).

^13^C NMR (CDCl_3_; δ, ppm) *endo*-isomer: 137.72, 132.19, 49.67, 45.20, 42.61, 38.05, 37.57, 33.10, 31.85; *exo*-isomer: 136.63, 46.18, 45.53, 42.04, 39.86, 37.67, 32.77, 32.60.

MS (EI) [C_9_H_13_Br]: 200, 202 g/mol (calculated mass 200.1, 202.1 g/mol).

#### 2.1.3. Synthesis of 5-(4-Bromobutyl)-2-norbornene (M4) [11]

Cyclopentadiene (5.1 g, 77.5 mmol), 6-bromohexene-1 (37.9 g, 232.4 mmol), and hydroquinone (1.6 mmol, 0.02 eq.) were added to an argonated ampoule. The ampoule was sealed and then heated at 170 °C for 120 h. The product was purified via successive distillations (0.04 Torr, 38–40 °C) to give 6.9 g of the desired product (yield: 39%).

^1^H NMR (CDCl_3_; δ, ppm): 6.13–5.89 (m, 2H), 3.46–3.35 (m, 2H), 3.40 (m, 2H), 2.81–2.71 (m, 2H), 2.07–1.90 (m, 1H), 1.90–1.76 (m, 3H), 1.42–1.36 (m, 2H), 1.35–1.18 (m, 2H), 1.15–1.01 (m, 2H), 0.49 (m, 1H).

^13^C NMR (CDCl_3_; δ, ppm) *endo*-isomer: 137.22, 132.40, 49.71, 45.49, 42.66, 38.72, 34.18, 33.98, 33.15, 32.53, 27.31; *exo*-isomer: 136.95, 136.40, 46.43, 45.35, 42.01, 38.75, 35.78, 34.14, 33.19, 27.56.

MS (EI) [C_11_H_15_Br]: 228, 230 g/mol (calculated mass 228.05, 230.05 g/mol).

### 2.2. General Procedures of Vinyl-Addition Polymerization (PM1–PM4)

#### 2.2.1. Polymerization in the Presence of the Single-Component Catalyst

An example of the synthesis of PM4 on the [SIMesPd(cinn)(MeCN)]^+^BARF^−^ catalyst at a monomer/Pd molar ratio of 4500/1 is given. The monomer M4 (4.85 g, 21.2 mmol) was dissolved in 91.6 mL of absolute dichloromethane, and the solution of the [SIMesPd(cinn)(MeCN)]^+^BARF^−^ catalyst in dichloromethane (9.4 mL, 4.7 × 10^−6^ mol, 5.0 × 10^−4^ M) was added to the solution of the monomer upon stirring. The stirring was continued for 2.5 h at 45 ℃. The procedure was carried out in the air. The polymerization mixture was poured into CH_3_OH containing 5% MeCN. The polymeric powder was filtered, washed with CH_3_OH, and dried under a reduced pressure. The polymer was reprecipitated by a CH_3_OH-CH_3_CN mixture from its solution in CHCl_3_, and dried under a reduced pressure (2.23 g, yield: 46%). M_n_ = 9.1 × 10^5^, M_w_/M_n_ = 2.1.

#### 2.2.2. Polymerization in the Presence of the Three-Component Catalytic System

An example of the synthesis of PM4 on the SIPrPd(cinn)Cl/PCy_3_/NaBARF system at a monomer/Pd molar ratio of 4500/1 is given. The monomer M4 (4.00 g, 9.8 mmol) was dissolved in 37.5 mL of absolute dichloromethane and 7.4 mL of the solution of the catalytic system, consisting of SIPrPd(cinn)Cl (2.2 × 10^−3^ mmol, 2 × 10^−3^ M, 1 eq), NaBARF (9.8 mmol, 1 × 10^−2^ M, 5 eq.) and PCy_3_ (4.4 × 10^−3^ mmol, 5 × 10^−3^ M, 2 eq.). The stirring was continued for 1.5 h at 45 °C. The solution of the catalytic system was prepared in the Glovebox. The polymerization procedure was carried out in the air. The procedure for isolating the polymer is similar to that described above (1.60 g, yield: 40%). M_n_ = 1.39 × 10^6^, M_w_/M_n_ = 1.5.

^1^H NMR (CDCl_3_; δ, ppm): 3.55–3.22 (m, 2H, -CH_2_-Br), 2.68–0.17 (m, 15H).

^13^C NMR (CDCl_3_; δ, ppm): 55.54–30.59, 28.94–25.52.

*Polymer MP1*

^1^H NMR (CDCl_3_; δ, ppm): 4.053–2.90 (m, 2H, -CH_2_-Br), 2.88–0.17 (m, 9H).

^13^C NMR (CDCl_3_; δ, ppm): 54.27–40.52, 40.48–31.06.

*Polymer MP2*

^1^H NMR (CDCl_3_; δ, ppm): 3.73–3.19 (m, 2H, -CH_2_-Br), 3.17–0.28 (m, 11H).

^13^C NMR (CDCl_3_; δ, ppm): 54.98–35.20, 35.08–31.47.

### 2.3. Procedure for Copolymerization of M1–M4 Monomers

Monomers M1 (1500 eq., 0.17 M), M2 (1500 eq., 0.17 M), and M4 (1500 eq., 0.17 M) were mixed in a vial with the absolutated dichloromethane. Mesitylene (1500 eq.) as a standard was added to this mixture. The copolymerization was initiated by adding the catalytic mixture (0.121 mL) prepared in the absolutated dichloromethane and consisting of SIPrPd(cinn)Cl (1 eq., 3.6 × 10^−4^ M), NaBARF (5 eq., 1.8 × 10^−3^ M) and PCy_3_ (2 eq., 7 × 10^−4^ M). The copolymerization was carried out at 45 °C. Aliquots (~0.06–0.08 mL) were taken off and were precipitated with a MeOH-MeCN mixture (95/5 (*v/v*)). The polymer was separated, and the residue liquid solutions were used for the analysis. GLC chromatography was used to monitor the monomers’ conversion relative to mesitylene as the internal standard.

## 3. Results and Discussion

### 3.1. Vinyl-Addition Polymerization of 5-Bromoalkylnorbornenes

**Scheme 1:**
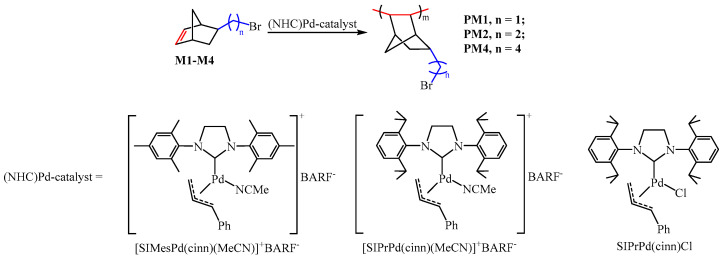
Vinyl-addition polymerization of 5-bromoalkylnorbornenes.

Little is known about the *homo*polymerization of bromoalkylnorbornenes. Mostly, Ni- and Pd-based systems are tested as catalysts [6,9,13], and the single-component Ni-catalyst, [Ni(C_6_F_5_)_2_(SbPh_3_)_2_], has turned out to be the most effective [12]. Polymerization of a bromoalkylnorbornene containing a long linker on this catalyst proceeded more smoothly and afforded polymers with moderate molecular weights, while the polymerization of the related monomers with shorter linkers gave only low-molecular-weight products. The molecular weights of such polymers were usually insufficient to cast free-standing and robust polymer films. Recently, it has been demonstrated that (NHC)Pd-based systems catalyze vinyl-addition polymerization of various functionalized norbornenes [14,15,16,17,18,19,20,21]. For instance, these systems showed good catalytic activity in polymerization of norbornenes with hydroxy [20,22], ether [21], ester [14,15,18,19,20,23,24], imide [14,16,17], silyl [14,19,20,24], and alkyl/alkenyl [19,20,22,24,25] functionality. At the same time, to the best of our knowledge, the polymerization of norbornene-type monomers with reactive halogen-containing groups over (NHC)Pd-catalysts has not yet been reported. Here, we investigated the catalytic behavior of the most active such catalysts in vinyl-addition polymerization of bromoalkylnorbornenes (M1–M4, Scheme 1). To assess their reactivity in the polymerization of M1–M4 and to evaluate the effect of the catalyst’s structure on the molecular weight characteristics of the resulting polymers, we selected both single- and three-component (NHC)Pd-systems. As single-component catalysts, the complexes [SIMesPd(cinn)(MeCN)]^+^BARF^−^ and [SIPrPd(cinn)(MeCN)]^+^BARF^−^ [14] were selected, while the systems consisting of SIPrPd(cinn)Cl, a phosphine (tricyclohexylphosphine (PCy_3_) or tri(*tert*-butyl)phosphine (P*^t^*Bu_3_)), and a cocatalyst (NaBARF, BARF^−^ —[(3,5-(CF_3_)_2_C_6_H_3_)_4_B]^−^) [15] were applied as three-component catalysts.

**Figure 3:**
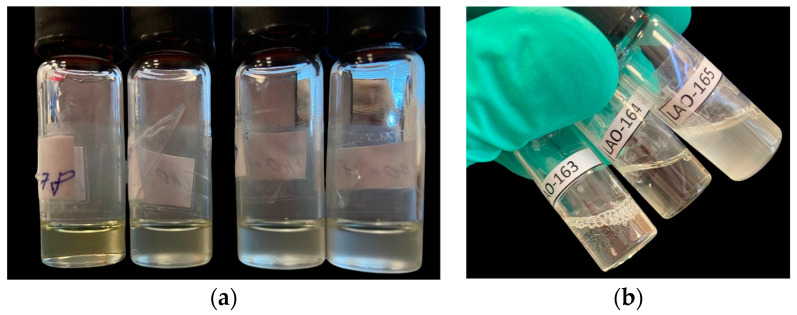
The photographs of the polymerization mixtures (**a**) in toluene (the monomer—M2, catalyst—SIPrPd(cinn)Cl/PCy_3_/NaBARF) and (**b**) in chlorobenzene and in chloroform (two bottom vials) and in toluene (the upper vial) (the monomer—M2, catalyst—[SIMesPd(cinn)(MeCN)]^+^BARF^−^).

Intriguing results were obtained when studying the polymerization of M1–M4 on these (NHC)Pd-catalysts. The single-component catalyst [SIMesPd(cinn)(MeCN)]^+^BARF^−^ showed very high activity in the polymerization of M2 and M4 monomers (Table 1). The polymers formed in moderate and good yields even at very high monomer/Pd molar ratios (the catalyst loadings were as low as 0.017 mol.%). The polymerization took place at mild temperatures (30–45 °C). The polymerization of M2 and M4 monomers in dichloromethane or in chloroform was homogenous, while the precipitation of the resulting polymers was observed during the polymerization in toluene or in chlorobenezene (Figure 3). Therefore, as the most suitable solvent for the polymerization, dichloromethane was chosen, which also afforded polymers with higher yields compared to chloroform or chlorobenzene (Table 1). The yields of polymers only slightly dropped with increasing monomer/[SIMesPd(cinn)(MeCN)]^+^BARF^−^ molar ratios, while the molecular weights of the polymers formed increased in most cases with the increase in monomer/Pd molar ratio (e.g., polymerization of M4, Table 1). This indicates the high stability of active catalytic Pd-centers in the polymerization mixtures. It is worth noting that if the [SIMesPd(cinn)(MeCN)]^+^BARF^−^ complex exhibited a very high activity in the homopolymerization of M2 and M4, then the related complex [SIPrPd(cinn)(MeCN)]^+^BARF^−^ (Scheme 1), which is also a single-component catalyst, did not catalyze the homopolymerization of these monomers (Table 1). This difference is likely due to steric hindrances of the larger NHC ligand (SIPr). Interestingly, the [SIMesPd(cinn)(MeCN)]^+^BARF^−^ catalyst provided soluble polymers of M4 and insoluble polymers of M2.

Therefore, the [SIMesPd(cinn)(MeCN)]^+^BARF^−^ complex efficiently catalyzed the polymerization of M2 and M4. However, this complex was unexpectedly inactive in the homopolymerization of M1. Only traces of a polymer were obtained as a result of homopolymerization of M1 on [SIMesPd(cinn)(MeCN)]^+^BARF^−^ catalyst at low monomer/Pd molar ratios (Table 1). The reason for such a behavior of the [SIMesPd(cinn)(MeCN)]^+^BARF^−^ complex is not clear. Nevertheless, we succeeded in showing that various 5-bromoalkylnorbornenes, including the M1 monomer, can be easily polymerized using a three-component catalytic (NHC)Pd-system, consisting of SIPrPd(cinn)Cl, tricyclohexylphosphine (PCy_3_) and an organoboron cocatalyst (NaBARF, Table 2, Scheme 1). Although the activity of this catalytic system was significantly lower compared to the [SIMesPd(cinn)(MeCN)]^+^BARF^−^ catalyst, it afforded soluble homopolymers based on all the studied monomers. Dichloromethane turned out to be a more suitable solvent for polymerization. The yields of polymers can be improved markedly by using lower monomer concentrations and increasing reaction times (Table 2).

**Figure 4:**
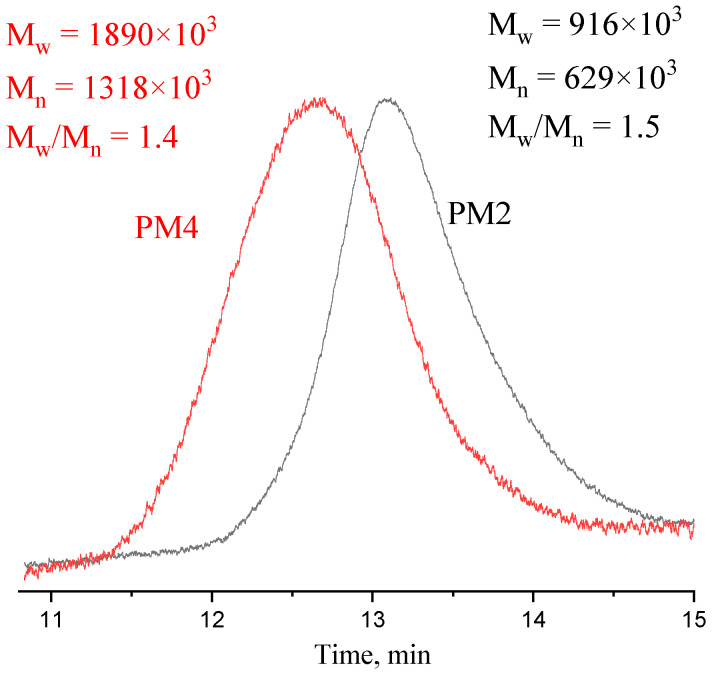
GPC curves of polymers PM2 and PM4 obtained in the presence of the three-component catalytic system (SIPrPd(cinn)Cl/NaBARF/PCy_3_).

**Figure 5:**
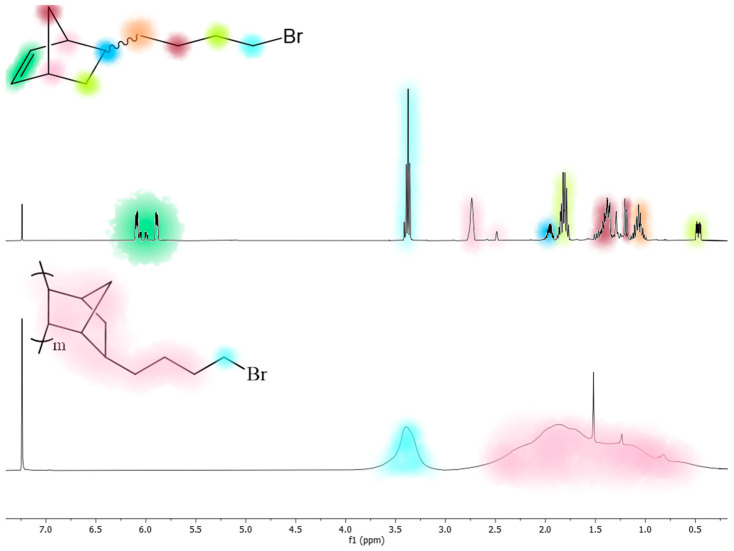
^1^H NMR spectra of M4 and vinyl-addition polymer obtained from M4 (PM4, the catalyst—[SIMesPd(cinn)(MeCN)]^+^BARF^−^ complex, CDCl_3_).

Interestingly, replacing tricyclohexylphosphine with a more sterically hindered tri(*tert*-butyl)phosphine in this catalytic system led to the disappearance of catalyst activity. Tuning the polymerization conditions (monomer/Pd molar ratio, monomer concentration, solvent) allowed us to obtain soluble high-molecular-weight polymers on the SIPrPd(cinn)Cl/PCy_3_/NaBARF system. For instance, employing this catalytic system, the homopolymers from M4 and M1 monomers were prepared with M_n_ up to 1.4 × 10^6^ and up to 1.2 × 10^5^, respectively. These values of M_n_ are more than ten times higher than previously published molecular weights of homopolymers based on these monomers [9,11]. It should be noted that GPC analysis is a relative method, and polystyrene and the synthesized polynorbornenes have different hydrodynamic radius. Nevertheless, earlier, we showed for several polynorbornenes that molecular weights (M_w_) determined via GPC analysis and molecular weights determined via the static light-scattering method are close (e.g., for vinyl-addition poly(5-ethylidene-2-norbornene) [26]). Therefore, taking into account our previous results, we assume that it is possible to assess the molecular weights of the polymers from 5-bromoalkylnorbornenes using GPC analysis. The determination of the true molecular weights of these polymers is in progress now. The resulting polymers have monomodal GPC curves (Figure 4), and the values of molecular weight distributions in most cases are less than 2.6, indicating that the polymerization occurred at active centers of one type. The structure and purity of the polymers were confirmed with ^1^H and ^13^C NMR spectroscopy (Figure 5 and Figures S1–S13).

**Figure 6:**
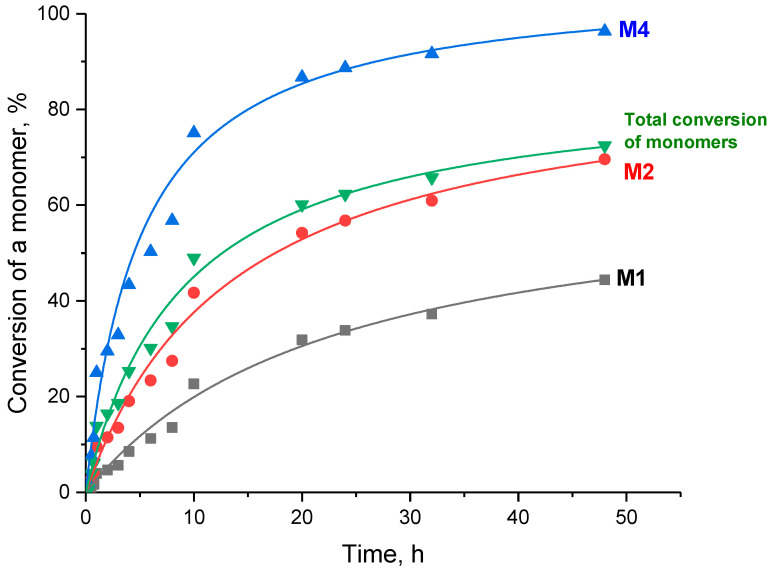
The dependence of the monomers’ conversions on time during the vinyl-addition copolymerization of the M1, M2, and M4 monomers (catalytic system—SIPrPd(cinn)Cl/PCy_3_/NaBARF, molar ratio of components in the catalytic system—SIPrPd(cinn)Cl/PCy_3_/NaBARF = 1/2/5, molar ratio of M1/M2/M4/Pd = 1500/1500/1500/1, the total monomers’ concentration—0.5 M, [M1] = [M2] = [M4] = 0.167 M, solvent CH_2_Cl_2_, 45 °C. The monomers’ conversions were assessed via GLC analysis using mesitylene as an internal standard).

The reactivity of monomers during vinyl-addition polymerization is usually determined by the size of the substituent in a monomer and/or by the presence of polar or reactive functionality in the substituent of a monomer. Taking into account the solubility and the values of molecular weight distributions of the resulting polymers, the interaction of the C(sp^3^)-Br bond with active catalytic Pd-species seems unlikely; thus, bromoalkyl groups in the studied monomers could be considered inert substituents. The monomer M1 contains a smaller substituent compared to the monomers M2 and M4. However, the reactivity of the M1 monomer was lower on both single- and three-component Pd-catalytic systems. This result is unexpected, and to exclude the influence of trace impurities on vinyl-addition polymerization, we assessed the relative reactivities of monomers M1–M4 in the copolymerization reaction (Figure 6). The monomer’s conversion was monitored using GLC analysis. Based on the obtained data, it is clear that the reactivity of M4 is indeed significantly higher than that of M1, while the reactivity of M2 was intermediate, located between the reactivities of M1 and M4, and was close to the total conversion of all three monomers. Therefore, the reactivity of a 5-bromoalkylnorbornene increases with the length of the linker between the norbornene motif and the bromine atom. Additionally, apparently, although there is no cleavage of the C-Br bonds in the polymerization of 5-bromoalkylnorbornenes, some interactions of the C-Br bond with the active Pd center probably take place when the monomer molecule is coordinated with Pd. This effect is more pronounced for 5-bromoalkylnorbornene, with a shorter linker namely for the M1 monomer.

### 3.2. The Basic Physico-Chemical Properties of the Prepared Vinyl-Addition Homopolymers Based on 5-Bromoalkylnorbornenes

**Figure 7:**
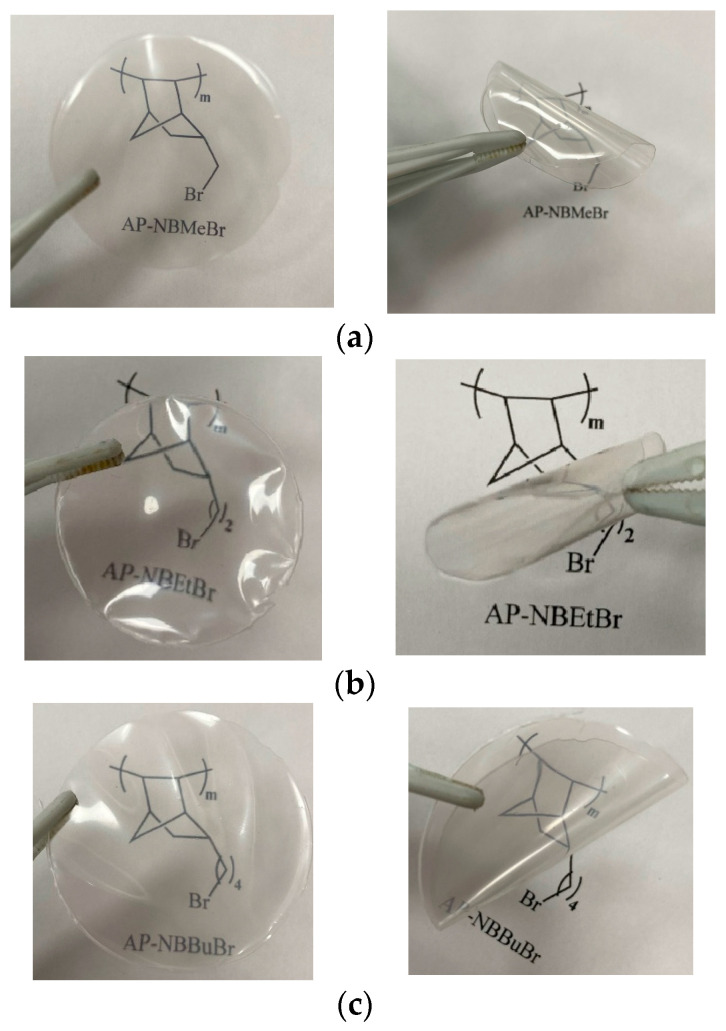
Images of thin films of vinyl-addition polymers: PM1 (**a**), PM2 (**b**), and PM4 (**c**) (the thickness of the films is ~100 μm and the diameter is 7 cm).

The prepared vinyl-addition polynorbornenes are well soluble in common organic solvents such as CHCl_3_ or tetrahydrofuran, and the polymers are insoluble in many other organic solvents (Table 3). Thanks to their high molecular weights and solubility in organic solvents, robust thin films of PM2 and PM4 polymers were easily prepared by casting from the corresponding solutions of the polymers in chloroform (Figure 7). The mechanical properties of the resulting polymers are typical of glassy polymers, possessing high T_g_ values (Table 4). The elongation at break and Young’s modulus for these polymers varied within the range of 4–16% and 540–1200 MPa, correspondingly (Table 5).

The densities of the synthesized vinyl-addition homopolymers based on 5-bromoalkylnorbornenes are in the range of 1.35–1.54 g/cm^3^ (Table 4), and these polymers possess moderate values of fractional free volume (FFV, 13–15%). A low porosity of PM2 and PM4 was also confirmed by the low-temperature nitrogen adsorption/desorption method. Thus, BET surface areas for these polymers are below 40 m^2^/g.

**Figure 8:**
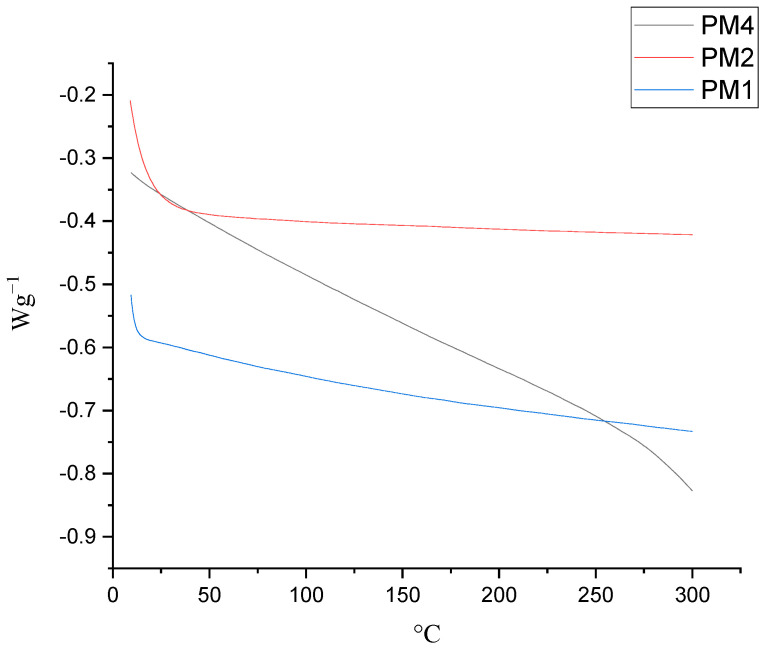
DSC curves of the prepared vinyl-addition polynorbornenes.

Vinyl-addition homopolymers based on 5-bromoalkylnorbornenes are glassy, and the values of T_g_ for these polymers are dropped down with the elongation of the linker in a monomer (Table 4, Figures S16–S18). The lowest T_g_ was for PM4, and the highest (above the onset of the decomposition) was for PM1. It is important to note that the T_g_s of these polymers could be determined by DMA analysis, while the corresponding effects were not observed in DSC curves (Figure 8).

**Figure 9:**
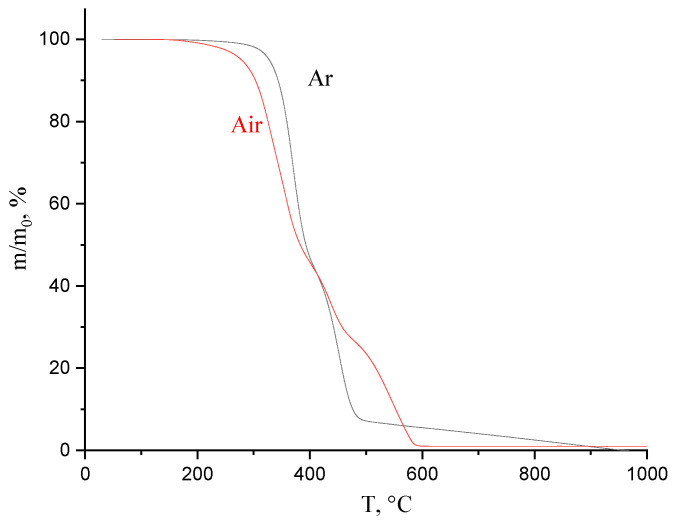
TGA curves of vinyl-addition PM4.

Despite the presence of labile C-Br bonds in the synthesized vinyl-addition polynorbornenes based on the M1–M4 monomers, the corresponding homopolymers showed good thermal stability (Figure 9, Figures S14 and S15). The temperatures of the decomposition (the loss of 5 wt.%) were above 260–280 °C in air and 290–330 °C in an inert atmosphere (Table 6). Minimal or zero char yields were at 1000 °C for the PM2 and PM4 polymers, indicating the complete decomposition of these polymers at high temperatures. At the same time, a noticeable char yield was observed for PM1 in argon (Figure S14), which can be explained by the less likely cleavage of side groups in the case of this polymer, and more efficient interchain crosslinking reactions upon heating.

**Figure 10:**
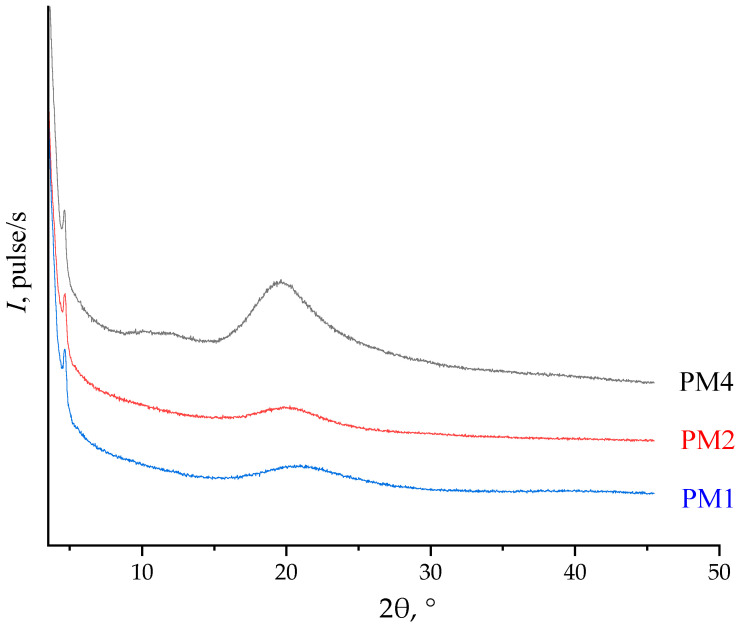
WAXD patterns for the studied vinyl-addition polymers from 5-bromoalkylnorbornenes (the peak at 2θ ~ 5° is due to the sample holder).

No crystallinity was detected in the vinyl-addition polymers prepared from 5-bromoalkylnorbornenes upon DSC and WAXD analysis. According to the WAXD study, the polymers have an amorphous nature (Figure 10, Table 7). There is only one broad peak in the WAXD patterns of the polymers with short linkers (PM1 and PM2), while the WAXD pattern of PM4 is represented by three broad peaks, showing a more ordered packing of polymer chains. The presence of broad peaks in the WAXD pattern of PM4 is probably due to more strong inter- and intra-molecular interactions. Previously, such behavior was observed for related polymers based on 5-alkylnorbornenes (e.g., 5-*n*-butylnorbornene and 5-*n*-hexylnorbornene [28]). The d-spaces for PM4 are larger compared to the corresponding d-values for PM1 and PM2 (Table 7). This may indirectly indicate a larger free volume in PM4 that is in good agreement with FFV values for these polymers (Table 4).

As a result of the high molecular weights and amorphous nature of the synthesized vinyl-addition polymers from 5-bromoalkylnorbornenes, we were able to obtain robust thin films from these homopolymers. This paves the way for new anion exchange membranes using such homopolymers as scaffolds.

## 4. Conclusions

The systematic study of vinyl-addition polymerization of 5-bromoalkylnorbornenes on (NHC)Pd-catalysts has opened up an opportunity to synthesize high-molecular-weight homopolymers from this class of monomers. Both single- and three-component Pd-systems can efficiently catalyze the polymerization of 5-bromoalkylnorbornenes, yielding soluble polymers with M_n_ in the range of 10^5^–10^6^. The reactivity of monomers dramatically depends on the length of the linker between the norbornene moiety and the bromine atom, while the size of the substituent in a norbornene-type monomer unexpectedly has a little effect. The monomer with the longest linker was the most active, while the polymerization of the similar monomer with the shortest linker was much slower, and required a higher catalyst loading. This clearly indicates an interaction of the C-Br bond of a monomer with the Pd-center when the monomer is coordinated with Pd through the norbornene double-bond. The resulting homopolymers have good film-forming capabilities, and durable thin films based on these polymers were effectively made. The synthesized vinyl-addition poly(5-bromoalkylnorbornenes) showed good thermal stability, and they are amorphous and glassy polymers. Their glass transition temperatures exceed 290 °C, allowing products based on them to function at high temperatures. Therefore, taking into account their amorphous nature, high glass transition temperatures, and good thermal stability combined with good film-forming properties, these homopolymers can be considered attractive precursors for new anion exchange membranes possessing improved ion conductivity.

## Figures and Tables

**Table 1:** The results of vinyl-addition homopolymerization of M2 and M4 monomers in the presence of the single-component catalyst, [SIMesPd(cinn)(MeCN)]^+^BARF^−^ complex.

Monomer (M)	M/Pd Molar Ratio	C(M), M	Reaction Time, h	T, °C	Solvent	Yield, %	M_w_∙10^−3^	M_n_∙10^−3^	M_w_/M_n_
M1	750	1	24	45	CH_2_Cl_2_	Traces	-	-	-
M2	1500	0.2	2.5	30	CH_2_Cl_2_	77	- ^a^	- ^a^	- ^a^
	1500	0.2	340	45	CH_2_Cl_2_	0 ^b^	-	-	-
	3000	0.2	2.5	30	CH_2_Cl_2_	72	- ^a^	- ^a^	- ^a^
	4500	0.2	2.5	30	CH_2_Cl_2_	68	- ^a^	- ^a^	- ^a^
	6000	0.2	2.5	30	CH_2_Cl_2_	66	- ^a^	- ^a^	- ^a^
	3000	0.2	2.5	30	PhCl	48	- ^a^	- ^a^	- ^a^
	3000	0.2	2.5	30	CHCl_3_	44	- ^a^	- ^a^	- ^a^
	3000	0.2	2.5	30	Toluene	Traces	- ^a^	- ^a^	- ^a^
M4	1500	0.2	500	45	CH_2_Cl_2_	0 ^b^	-	-	-
	4500	0.16	1.5	45	CH_2_Cl_2_	43	1662	969	1.7
	6000	0.16	1.5	45	CH_2_Cl_2_	39	1549	975	1.6
	3000	0.12	2.5	45	CH_2_Cl_2_	56	1712	766	2.2
	4500	0.12	2.5	45	CH_2_Cl_2_	46	1902	908	2.1
	6000	0.12	2.5	45	CH_2_Cl_2_	42	1834	1070	1.7
	3000	0.08	2	45	CH_2_Cl_2_	62	1629	637	2.6
	4500	0.08	2	45	CH_2_Cl_2_	55	1731	1016	1.7
	6000	0.08	2	45	CH_2_Cl_2_	50	1711	922	1.9

^a^—insoluble polymer was isolated; ^b^—[SIPrPd(cinn)(MeCN)]^+^BARF^−^ was used as a single-component catalyst.

**Table 2:** The results of the vinyl-addition homopolymerization of M1–M4 monomers in the presence of the three-component catalytic system (SIPrPd(cinn)Cl complex, PCy_3_ and NaBARF) ^a^.

Monomer (M)	M/Pd Molar Ratio	C(M), M	Reaction Time, h	T, °C	Solvent	Yield, %	M_w_∙10^−3^	M_n_∙10^−3^	M_w_/M_n_
M1	500	0.5	24	45	CH_2_Cl_2_	55	80	40	2.0
	1000	1	18	45	CH_2_Cl_2_	Traces ^b^			
	1000	0.5	24	45	CH_2_Cl_2_	35	243	87	2.8
	1500	1	18	45	CH_2_Cl_2_	40	311	124	2.5
	1500	0.2	6	45	Toluene	Traces	48	15.7	3.1
	1500	0.2	6	45	CH_2_Cl_2_	Traces	72	20	3.6
	2000	0.5	18	45	CH_2_Cl_2_	11	98	38	2.5
	2000	1	18	45	CH_2_Cl_2_	19	302	106	2.9
	3000	1	24	45	CH_2_Cl_2_	22	372	112	3.3
	4500	1	24	45	CH_2_Cl_2_	18	293	109	2.7
M2	1500	0.2	24	45	CH_2_Cl_2_	48	815	216	3.8
	3000	0.2	24	45	CH_2_Cl_2_	17	567	196	2.9
	4500	0.2	24	45	CH_2_Cl_2_	Traces	455	158	2.9
	6000	0.2	24	45	CH_2_Cl_2_	Traces	407	104	3.9
	1500	0.2	2.3	45	Toluene	62	916	629	1.5
	3000	0.2	2.3	45	Toluene	15	- ^c^	- ^c^	- ^c^
M4	1500	0.2	1.5	45	CH_2_Cl_2_	68	1571	642	2.4
	3000	0.2	1.5	45	CH_2_Cl_2_	53	1930	1245	1.6
	4500	0.2	1.5	45	CH_2_Cl_2_	40	2027	1393	1.5
	6000	0.2	1.5	45	CH_2_Cl_2_	32	1890	1318	1.4
	1500	0.15	2.5	45	CH_2_Cl_2_	72 ^d^	- ^f^	- ^f^	- ^f^
	1500	0.1	2.5	45	CH_2_Cl_2_	76 ^e^	- ^f^	- ^f^	- ^f^
	1500	0.2	2	45	Toluene	69	2013	1179	1.7
	3000	0.2	2	45	Toluene	43	1439	966	1.5

^a^—the composition of the catalyst system was 1 eq. of SIPrPd(cinn)Cl complex, 2 eq. of Pcy_3_, and 5 eq. of NaBARF; ^b^—tri(*tert*-butyl)phosphine was used instead of PCy_3_; ^c^—insoluble polymer was isolated; ^d^—the reaction mixture was diluted twofold with dichloromethane after 1 h; ^e^—dichloromethane (60% from the volume of the initial reaction mixture) was added to the reaction mixture after 1.5 h; ^f^—the molecular weights of the resulting polymers were not analyzed.

**Table 3:** The solubility of vinyl-addition homopolymers obtained from bromoalkylnorbornenes.

Polymer	Acetone	CH_2_Cl_2_	DMSO	DMF	CHCl_3_	C_6_H_6_	Et_2_O	THF	*n*-Hexane	PhCH_3_	CH_3_CN	PhCl
PM1	−	−	−	−	+	−	−	+	−	−	−	x
PM2	−	−	−	−	+	−	−	+	−	−	−	x
PM4	−	+	−	−	+	−	−	+	−	−	−	+

“+”—soluble, “−”—insoluble, “x”—swelling.

**Table 4:** Densities and FFV values of vinyl-addition homopolymers based on 5-bromoalkylnorbornenes ^a^.

Polymer	Density, g/cm^3^	FFV, %	T_g_, °C
PM1	1.538	13.8	>T_d_
PM2	1.475	13.3	304
PM4	1.357	14.7	291

^a^—the polymer’s density was determined via hydrostatic weighing; FFV values were evaluated using Bondi’s method [27].

**Table 5:** The Young’s modulus, tensile strength and elongation at break values of the synthesized vinyl-addition homopolymers.

Polymer	Tensile Strength, σ, MPa	Elongation at Break, ε, %	Young’s Modulus, E, MPa
PM1	10	5	1237
PM2	18	4	1008
PM4	9	16	544

**Table 6:** Thermal properties of the synthesized vinyl-addition polynorbornenes.

Polymer	T_d._, ^a^ °C	
	Ar	Air
PM1	287	262
PM2	333	271
PM4	328	278

^a^—temperature corresponding to 5% weight loss of the sample.

**Table 7:** The distances (*d*, Å) calculated based on WAXD data for the studied vinyl-addition polymers from 5-bromoalkylnorbornenes ^a^.

Polymer	*2θ*_1_, °	*d*_1_, Å	*2θ*_2_, °	*d*_2_, Å	*2θ*_3_, °	*d*_3_, Å
PM1	21.6	4.1	-	-	-	-
PM2	20.3	4.4	-	-	-	-
PM4	11.2	7.9	19.5	4.6	23.3	3.8

^a^—Bragg’s distance: *d* = λ/2sin(θ); λ = 1.54 Å.

## Data Availability

NMR spectra, TGA and DMA curves of the monomers and the polymers are available free of charge as a file within the Supplementary Materials.

## Supplementary Materials

The following supporting information can be downloaded at: https://www.mdpi.com/article/10.3390/polym15224444/s1, Figures S1–S8: NMR spectra of monomers; Figures S9–S13: NMR spectra of the polymers; Figures S14–S15: TGA curves of the polymers; Figures S16–S18: DMA curves of the polymers. References [29,30,31] are cited in the supplementary materials.
