# Supplementary material for: Vinyl-Addition Homopolymeization of Norbornenes with Bromoalkyl Groups
Source: Polymers (Basel). 2023 Nov 17;15(22):4444. doi: 10.3390/polym15224444 (PMC10674773; doi:10.3390/polym15224444)
Supplement: Supplementary file 1 [file polymers-15-04444-s001.zip › polymers-2703375-supplementary.pdf]

# Supplementary Material

# Vinyl-Addition Homopolymeization of Norbornenes with Bromoalkyl-Groups

**Artyom O. Lunin, Fedor A. Andreyanov, Igor S. Makarov and Maxim V. Bermeshev ***

A.V. Topchiev Institute of Petrochemical Synthesis, RAS, 29 Leninskiy Pr., 119991 Moscow, Russia; lunin@ips.ac.ru (A.O.L.); andreyanov@ips.ac.ru (F.A.A.); makarov@ips.ac.ru (I.S.M.)
* Correspondence: bmv@ips.ac.ru; Tel.: +7-495-647-5927 (ext. 379)

## *Table of contents*

1. Materials and methods ........ 2
2. NMR spectra of the monomers ........ 3
3. NMR spectra of the synthesized vinyl-addition homopolymers ........ 7
4. TGA curves of the synthesized vinyl-addition homopolymers ........ 10
5. DMA curves of the synthesized vinyl-addition homopolymers ........ 11
References ........ 13

# 1. Materials and methods

*Materials*

Dicyclopentadiene, allyl bromide, 4-bromobutene-1, 6-bromohexene-1, hydroquinone, NaBARF and $PCy_3$ were purchased from commercial suppliers (Sigma-Aldrich) and used as received unless otherwise noted. Methylene chloride, toluene, and chlorobenzene were distilled over $CaH_2$ under argon atmosphere and stored over molecular sieves (4 Å). Complexes $[SIMesPd(cinn)(MeCN)]^+BARF^-$, $[SIPrPd(cinn)(MeCN)]^+BARF^-$, and SIPrPd(cinn)Cl were prepared according to the procedures described earlier [1-4].

*Physico-chemical characterization*

NMR-spectra were recorded on a Bruker Ascend 400 spectrometer at 400.1 MHz ($^1H$) and at 100.6 MHz ($^{13}C$). Chemical shifts (δ) are reported in parts per million (ppm) relative to the reference (residual $CHCl_3$ signal) for $^1H$ and $^{13}C$ NMR spectra. Each sample of a polymer for NMR analysis was dissolved in $CDCl_3$ up to a concentration of 10%.

Gel-permeation chromatography (GPC) analysis of the polymers was performed on a Waters system with a differential refractometer (Chromatopack Microgel-5; eluent - chloroform; flow rate - 1 ml/min). The molecular weights and polydispersity were calculated by a standard procedure relative to monodispersed polystyrene standards.

Calorimetric measurements were conducted using a "Mettler" TA-4000 differential scanning calorimeter (Giesen, Germany) at a heating rate of 20°C/min under argon. TGA measurements were carried out on "TGA/DSC 1" (Mettler Toledo) in argon and in air at the heating rate of 10°C/min from 30 to 1000°C.

The glass transition temperature (DMA) was determined by the position of the maximum of the mechanical loss angle tangent, the temperature dependence of which was obtained on a Discovery HR-2 rotary rheometer (TA Instruments, USA) in the mode of periodic stretching of the sample film at a frequency of 1 Hz and a temperature rise rate of 5°C/min.

Wide-angle X-ray diffraction (WAXD) data were obtained using a two-coordinate AXS detector (Bruker, Bremen, Germany) and Cu K$\alpha$ emission (wavelength of 0.154 nm).

The density of polymer films was calculated by the hydrostatic weighing method according to the following procedure: a sample of the film was weighed on an analytical balance ($m_{dry}$). A thin copper wire was hung on a beam over the balance. The sample was fixed on the lower end of the wire, immersed in the beaker with methanol placed on the balance, and weighed ($m_1$). Then the sample was withdrawn and the free wire end immersed in methanol was weighed ($m_2$). The density of the film sample was calculated by the formula: $d = d_S\ m_{dry}/(m_{dry} – (m_1 – m_2))$, where $d_S = 0.791$ g/ml is the density of methanol.

Film mechanical testing was carried out on A I1140M-5-01-1 universal tensile testing machine (Tochpribor-KB, Ivanovo, Russia), and the ASTM D638 method was used for film mechanical testing. Dog-bone tensile specimens (ASTM standard D1708-96, 22 × 5 mm) were prepared by punching the films from a stainless steel die. The data is presented as the mean ± standard deviation (SD) and median with an interquartile range. A one-way ANOVA followed by Dunnett's multiple comparisons test was performed using Sigma Stat 3.5 (Systat Software, San Jose, CA, USA). Values of $p < 0.05$ were considered statistically significant.

The characteristics of porous structure of polymers were determined by nitrogen (77 K) adsorption using a Gemini VIIt analyzer (Micromeritics Instruments Corporation). All the samples were degassed at 100°C at 25-50 mTorr for 10 h before measurements. Surface area was calculated by the BET equation. The calculations of $S_{BET}$ were performed in the range of relative pressure ($p/p_0$) of 0.03–0.15.

GLC analysis was performed on the chromatograph "Kristall 5000" (Chromatek, Russia) with a capillary column SGE Analytical Science (Australia) and a flame ionisation detector (detector temperature 320°C, hydrogen flow rate: 25 ml·min$^{-1}$, air flow rate: 250 ml·min$^{-1}$, nitrogen flow rate 25 ml·min$^{-1}$ ). Column parameters: thermostat temperature 40°C, carrier gas pressure 71.519 kPa, carrier gas flow 2.5 ml·min$^{-1}$, carrier gas velocity 40 cm·sec$^{-1}$, discharge flow 30 ml·min$^{-1}$. Evaporator temperature: 250°C.

*Film preparation*

The polymer films were made by casting a polymer solution in chloroform at the concentration of 5 wt.%. The solution was poured into a steel cylinder with a diameter of 7 cm and a stretched cellophane bottom. The solvent was allowed to evaporate slowly at room temperature to yield the desired polymer film. After the formation of the films, the cellophane was detached, and the films were dried under vacuum at room temperature to a constant weight. A thermal treatment was not applied. The thickness of the films formed was ranged between 90-120 μm.

## 2. NMR spectra of the monomers

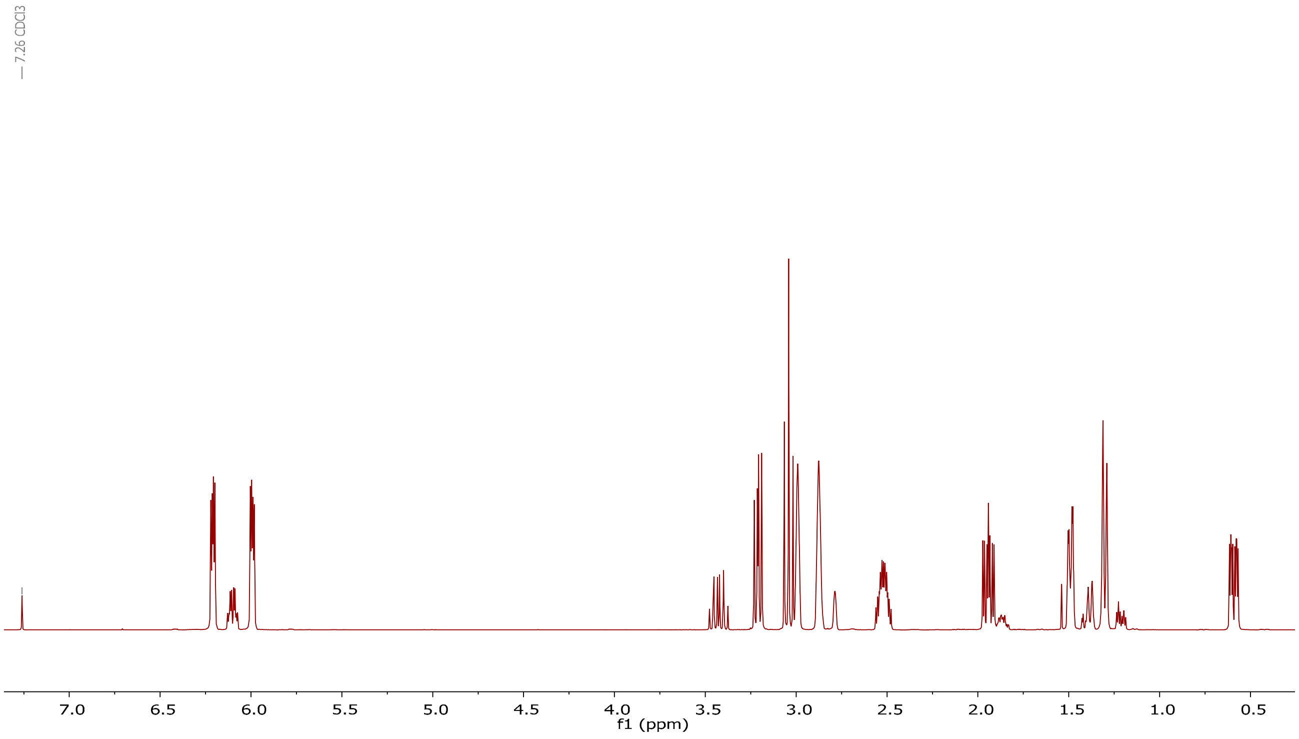


**Figure S1.** $^{1}H$ NMR spectrum of **M1** ($CDCl_3$).

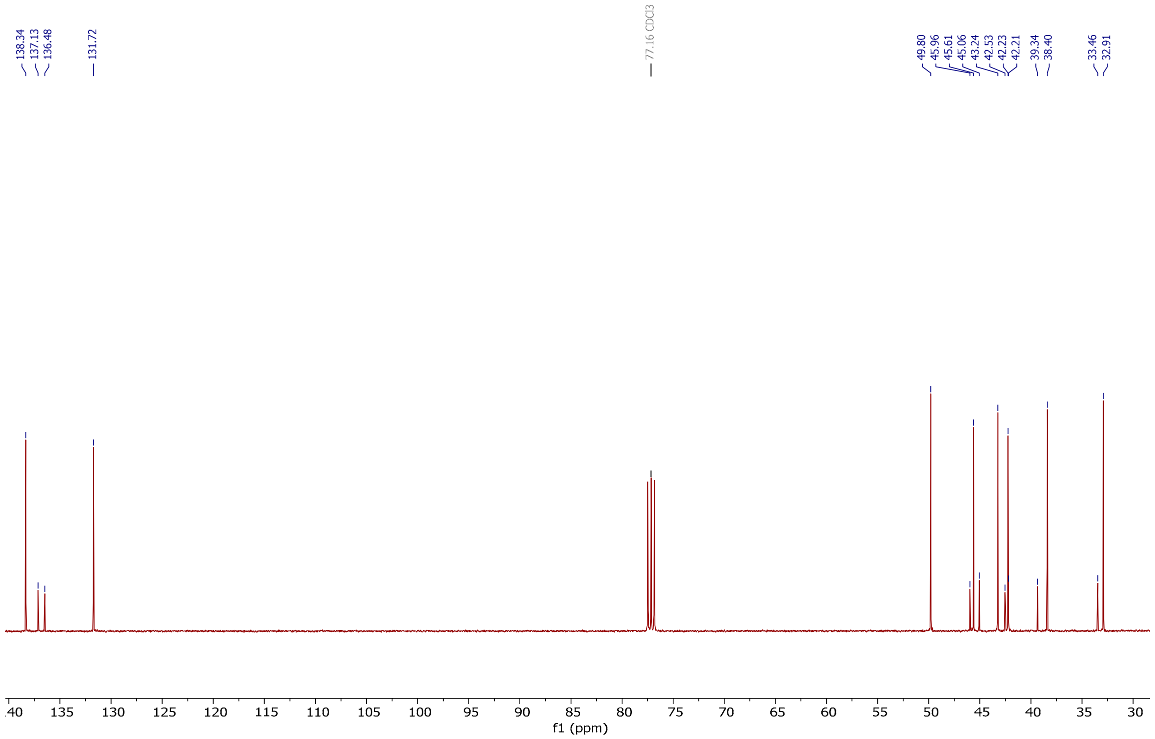


**Figure S2.** $^{13}C$ NMR spectrum of **M1** ($CDCl_3$).

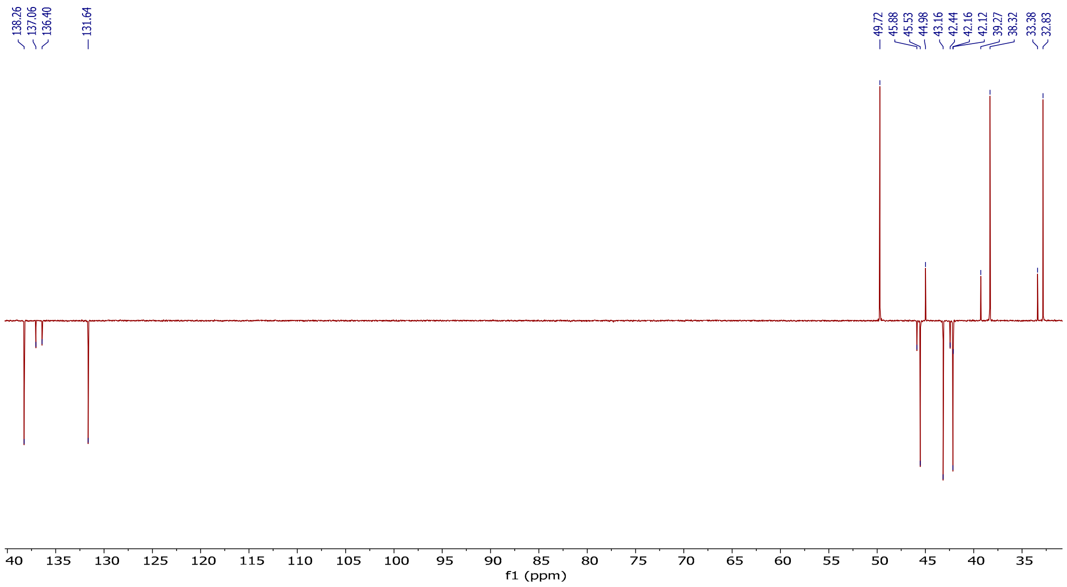


**Figure S3.** $^{13}$C NMR (DEPT 135) spectrum of **M1** ($CDCl_3$).

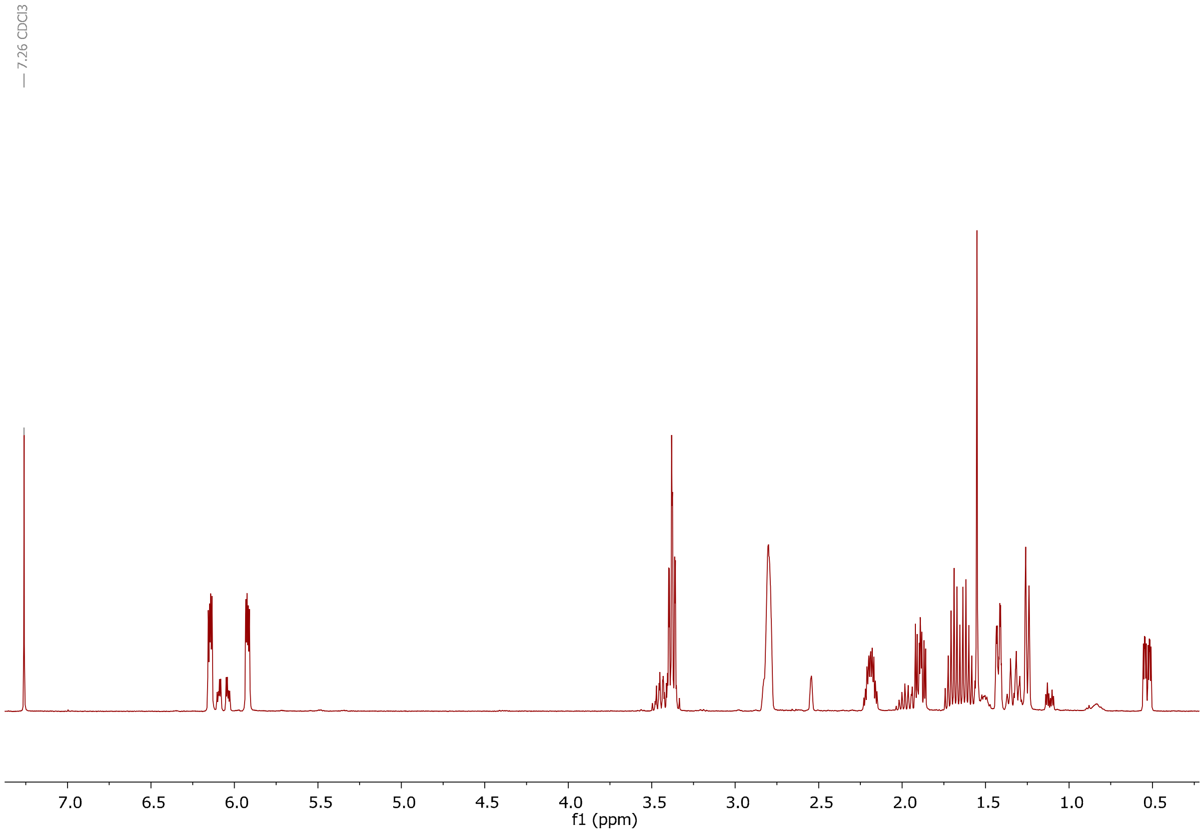


**Figure S4.** $^{1}$H NMR spectrum of **M2** ($CDCl_3$).

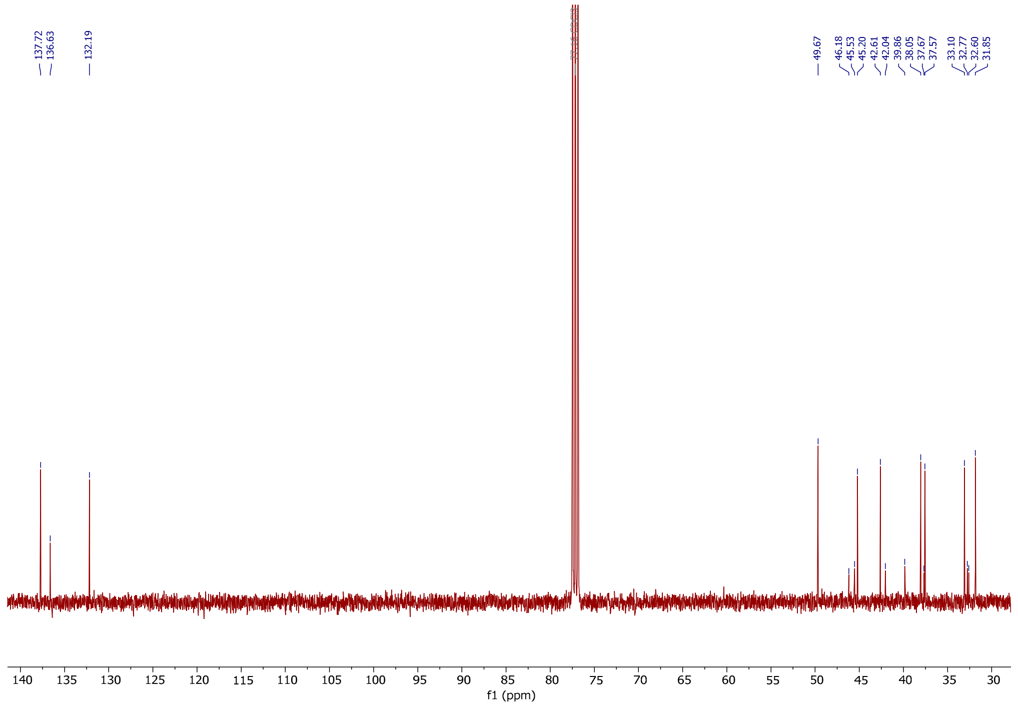


**Figure S5.** $^{13}C$ NMR spectrum of **M2** ($CDCl_3$).

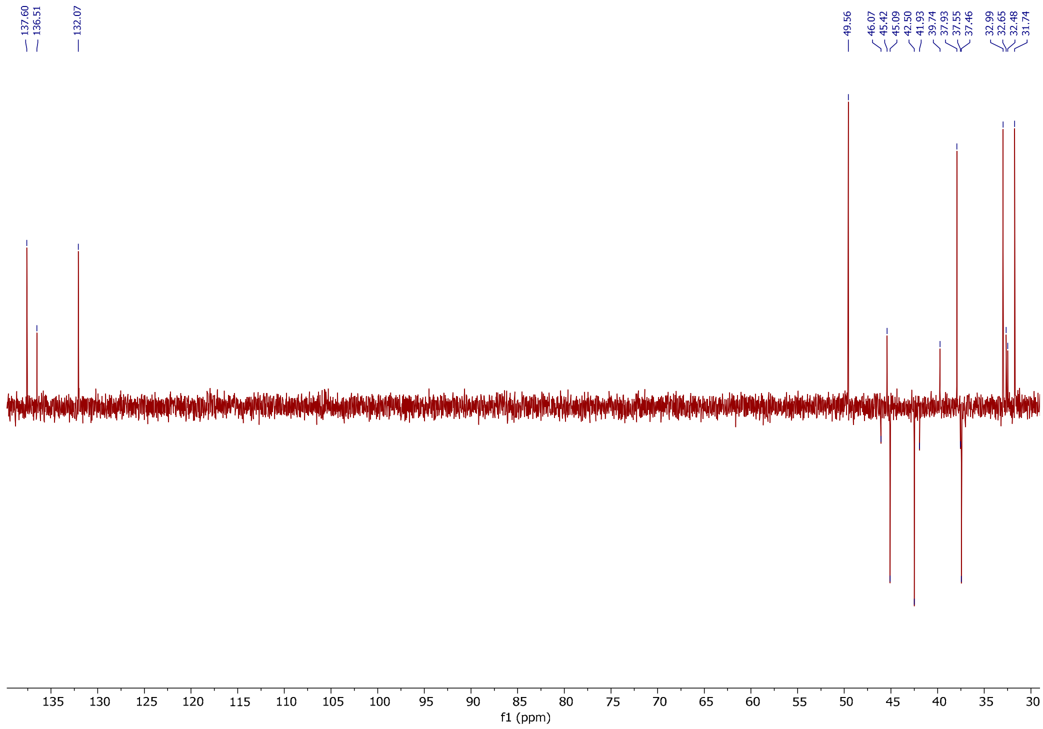


**Figure S6.** $^{13}C$ NMR (DEPT 135) spectrum of **M2** ($CDCl_3$).

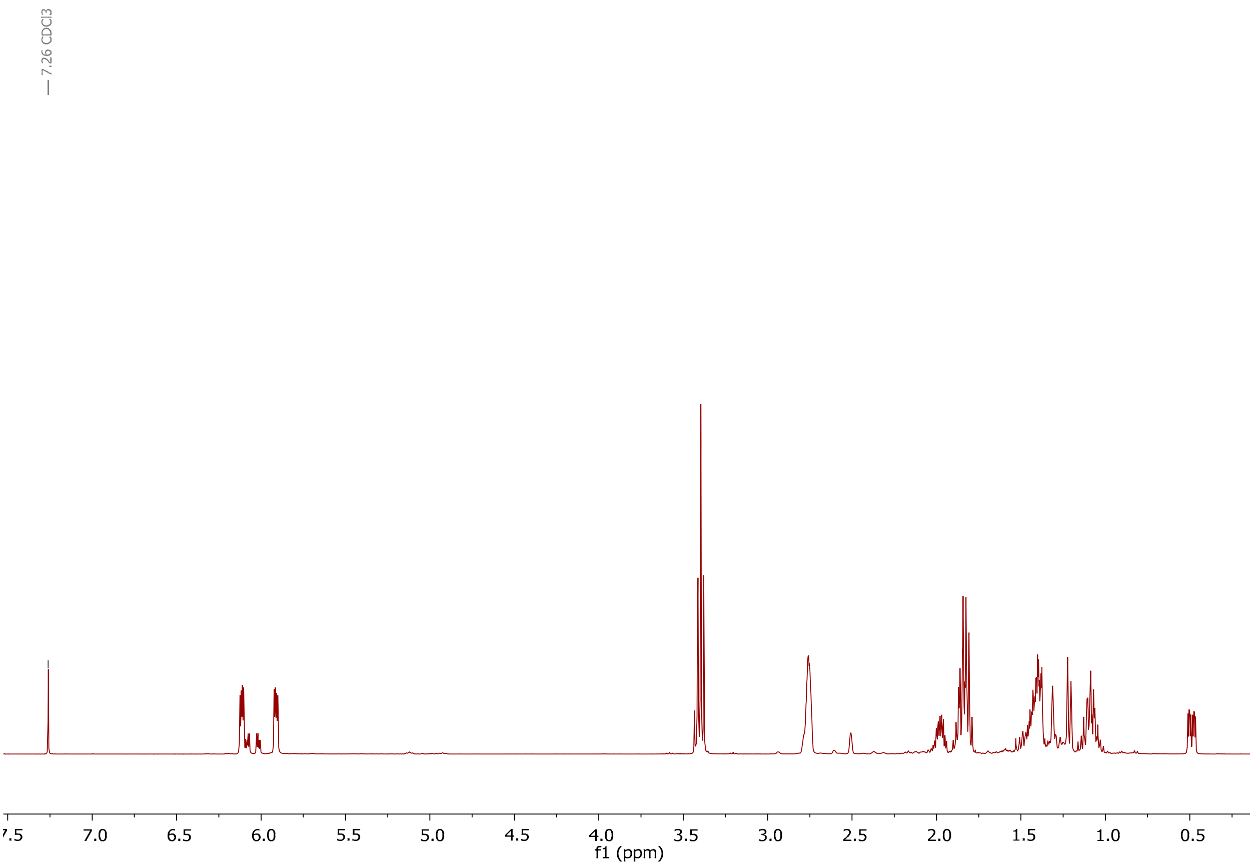


**Figure S7.** $^{1}H$ NMR spectrum of **M4** ($CDCl_3$).

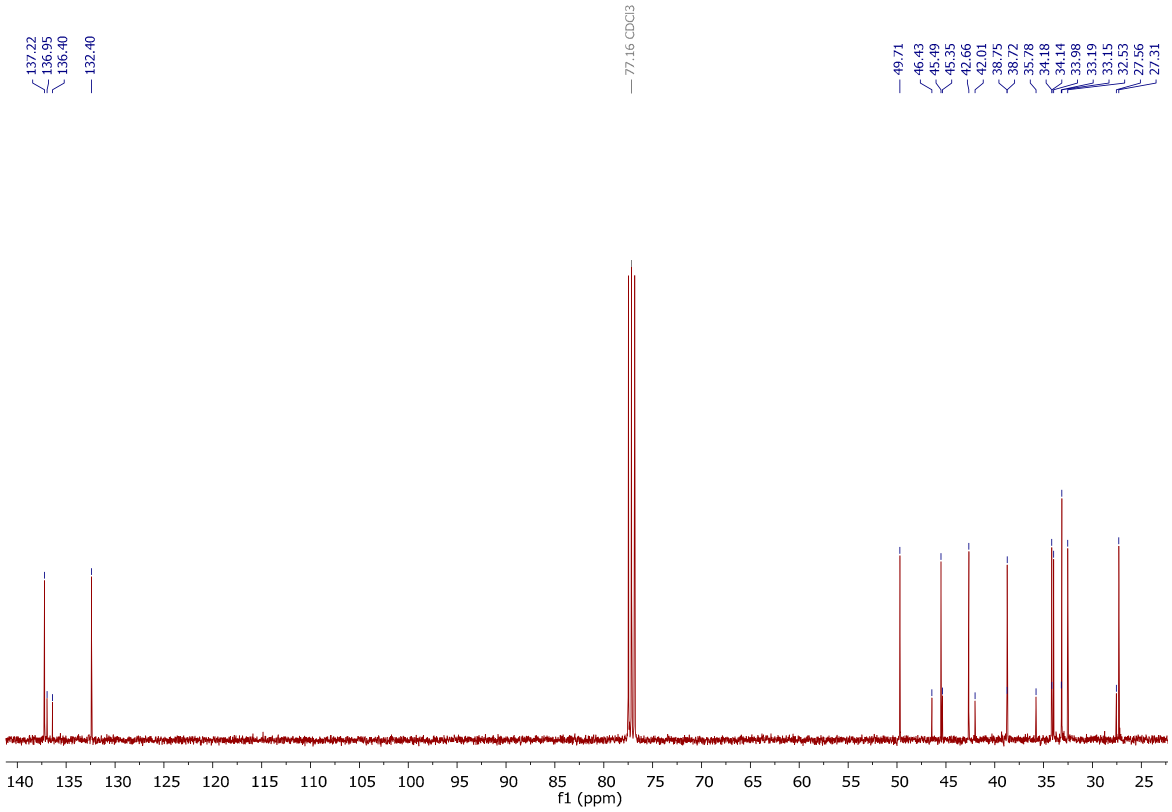


**Figure S8.** $^{13}C$ NMR spectrum of **M4** ($CDCl_3$).

## 3. NMR spectra of the synthesized vinyl-addition homopolymers

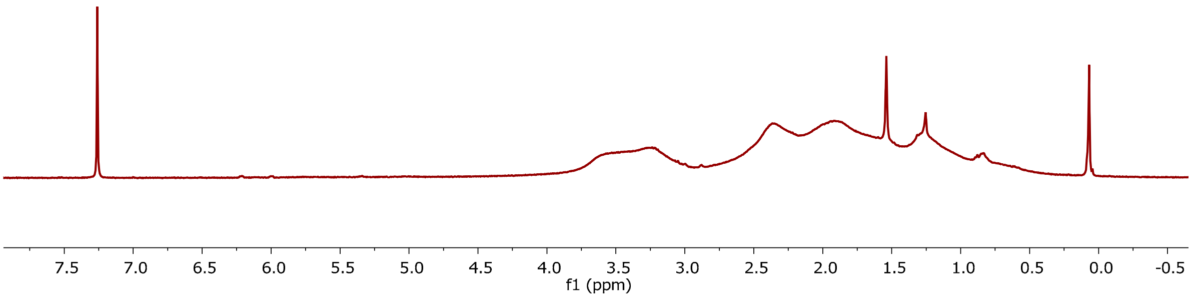


(a)

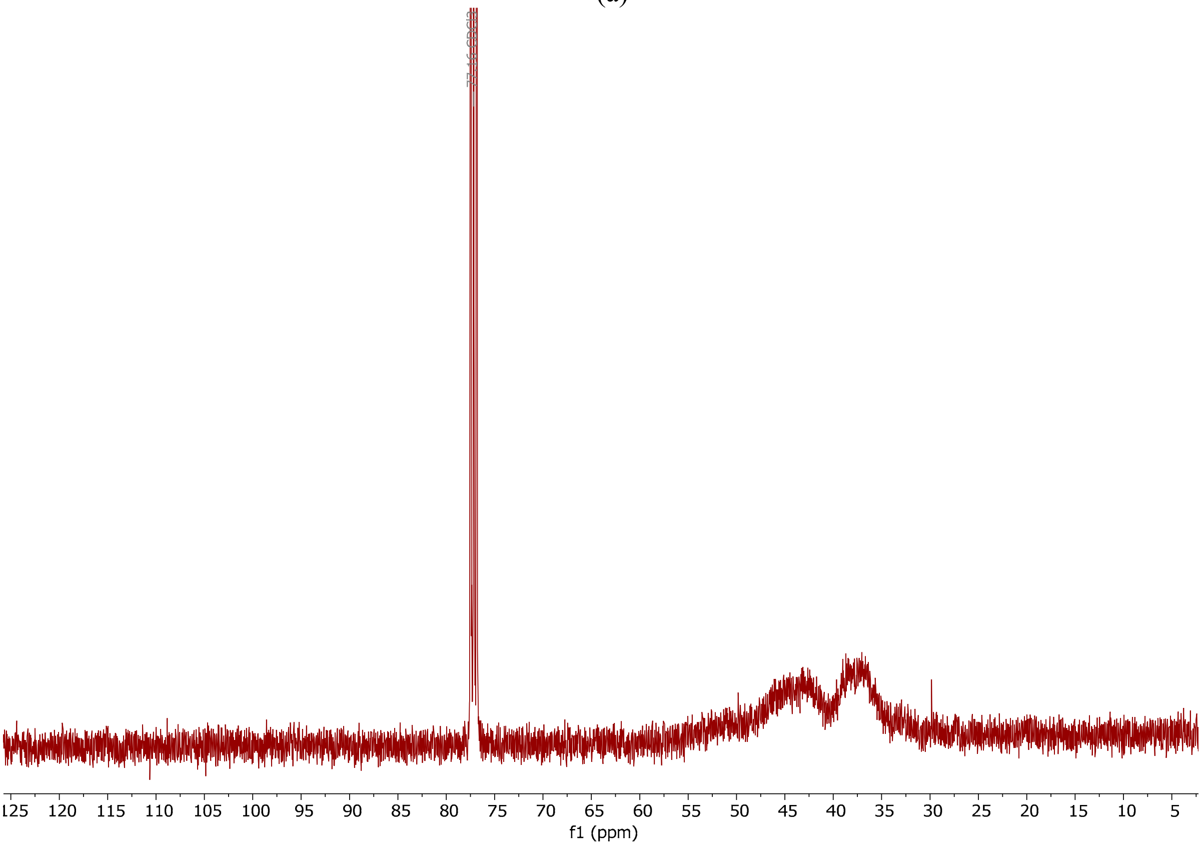


(b)

**Figure S9.** $^{1}H$ NMR spectrum (a) and $^{13}C$ NMR spectrum (b) of **PM1** ($CDCl_3$).

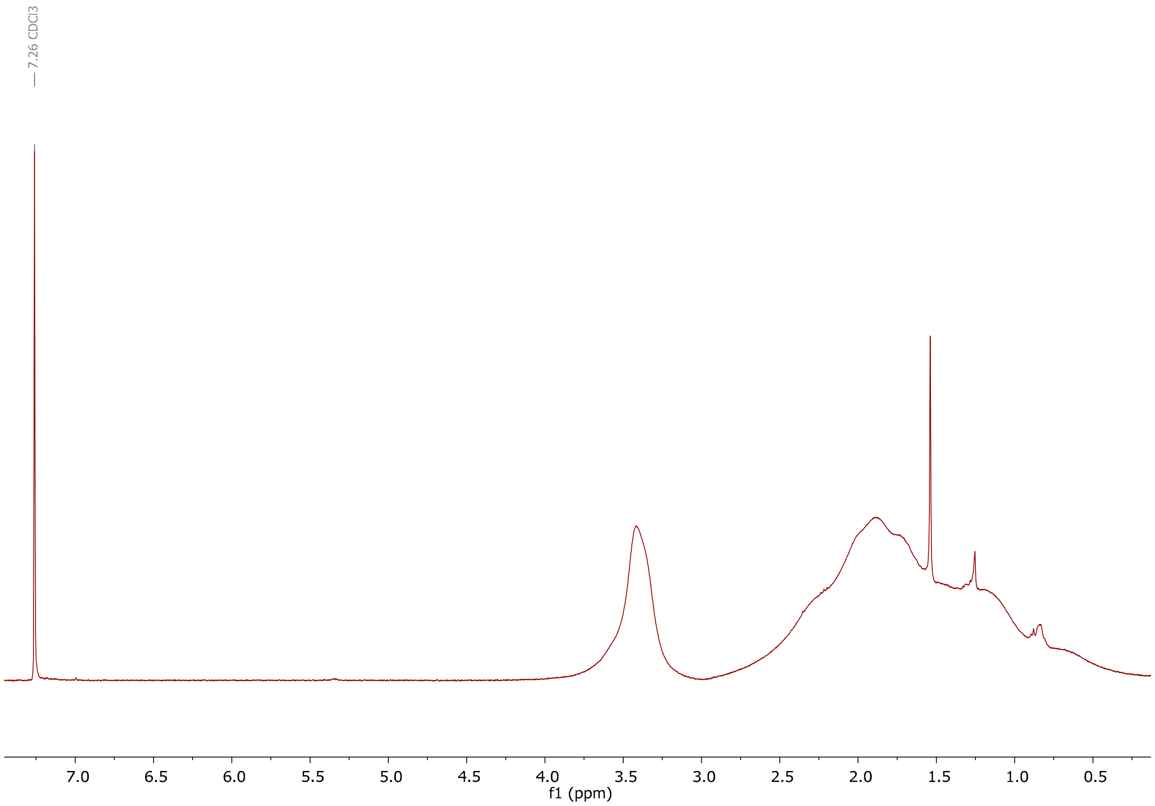


**Figure S10.** $^{1}H$ NMR spectrum of **PM2** ($CDCl_3$).

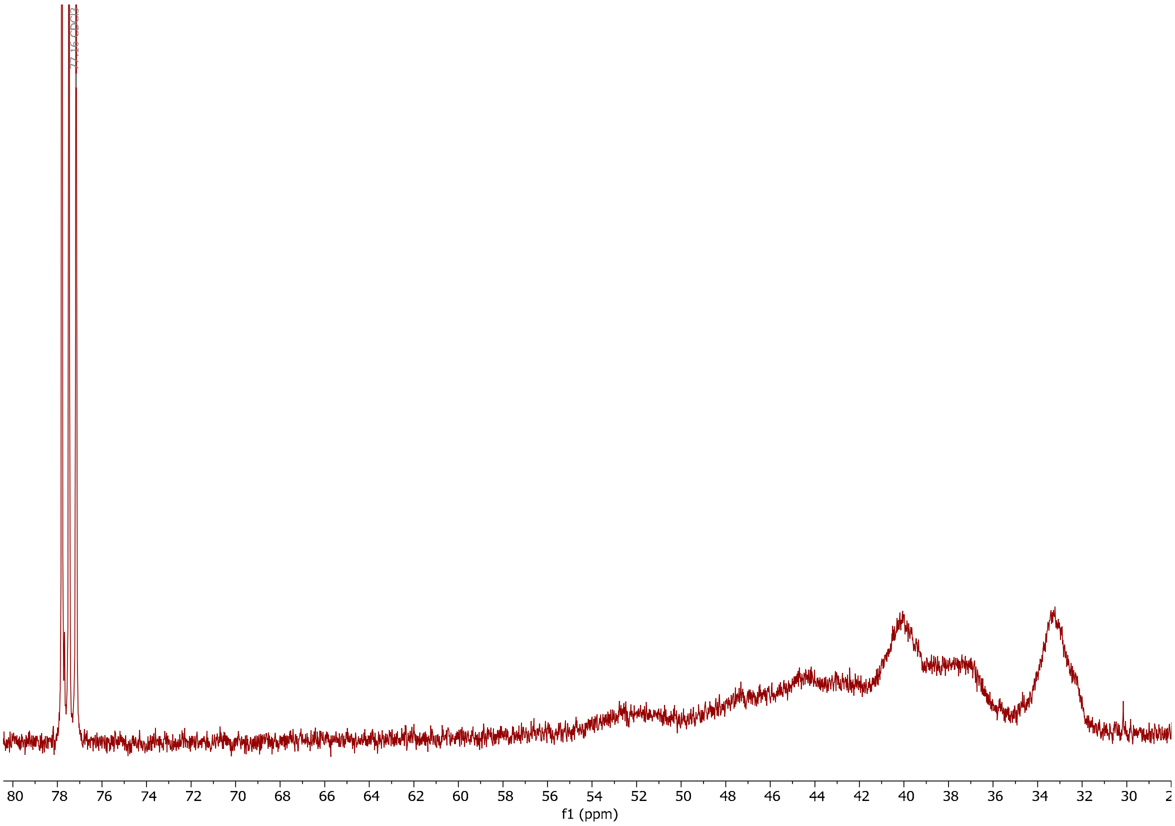


**Figure S11.** $^{13}C$ NMR spectrum of **PM2** ($CDCl_3$).

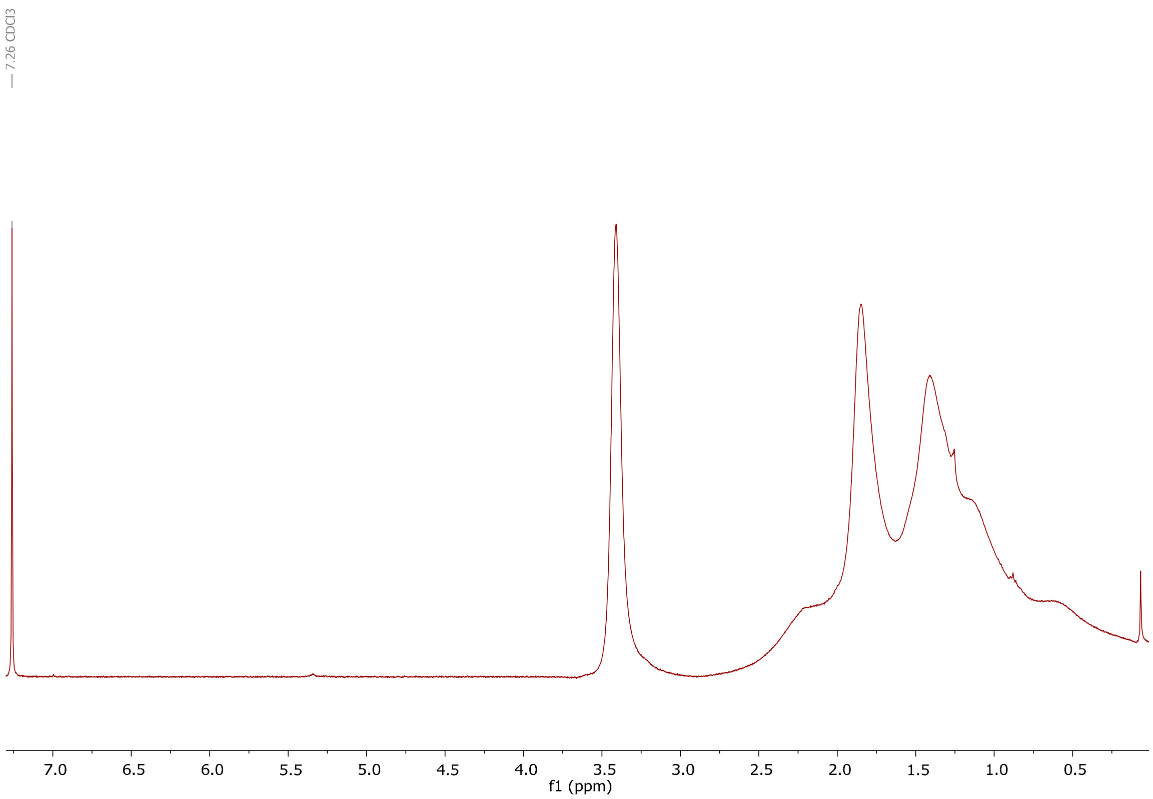


**Figure S12.** $^1$H NMR spectrum of **PM4** ($CDCl_3$).

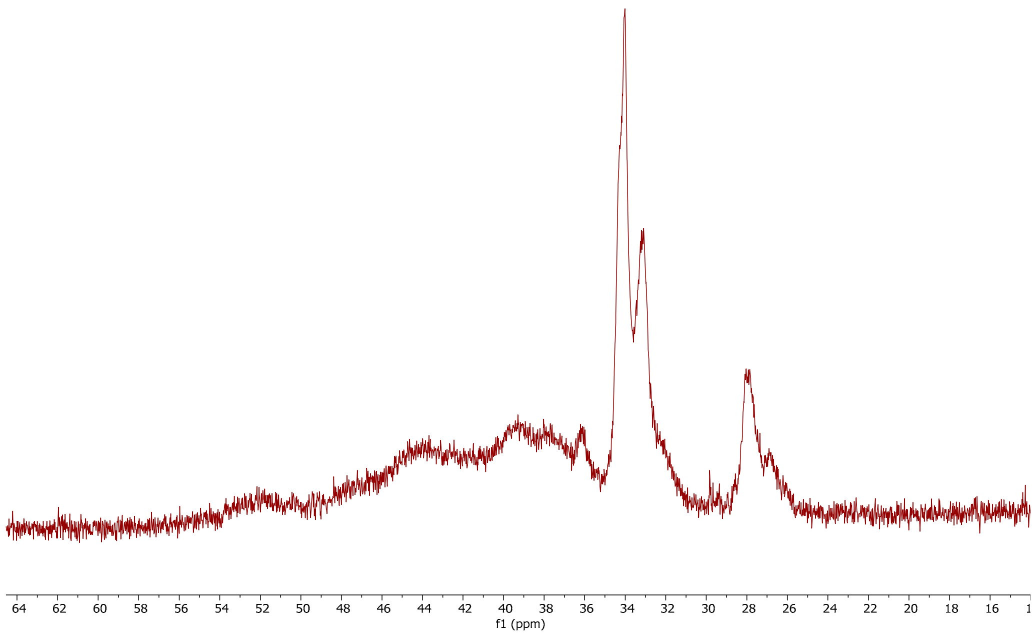


**Figure S13.** $^{13}$C NMR spectrum of **PM4** ($CDCl_3$).

## 4. TGA curves of the synthesized vinyl-addition homopolymers

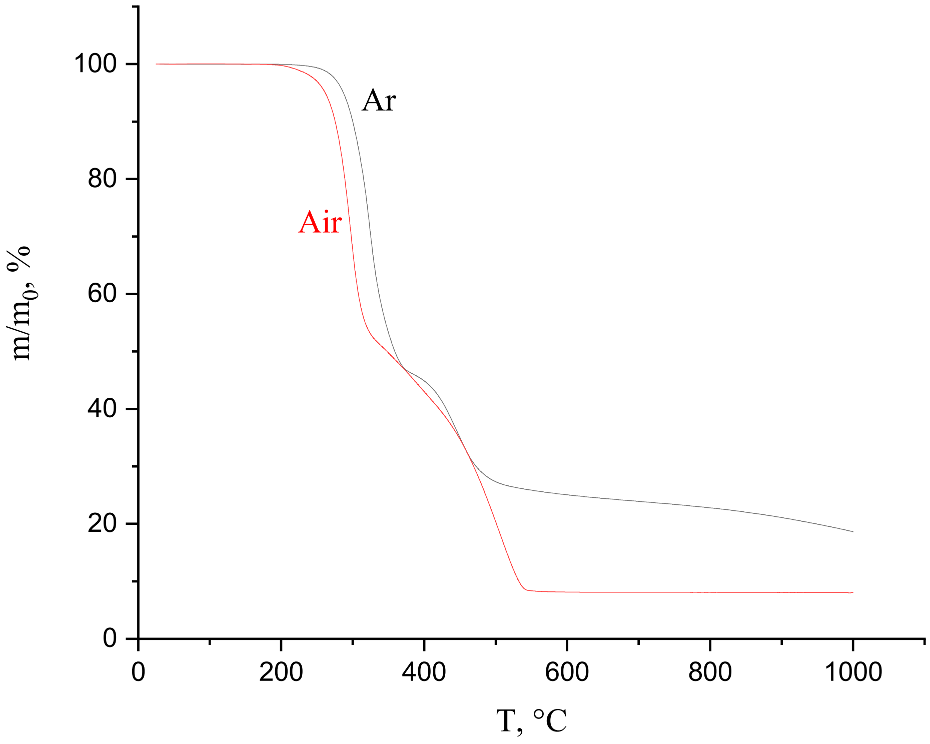


**Figure S14.** TGA curves for **PM1.**

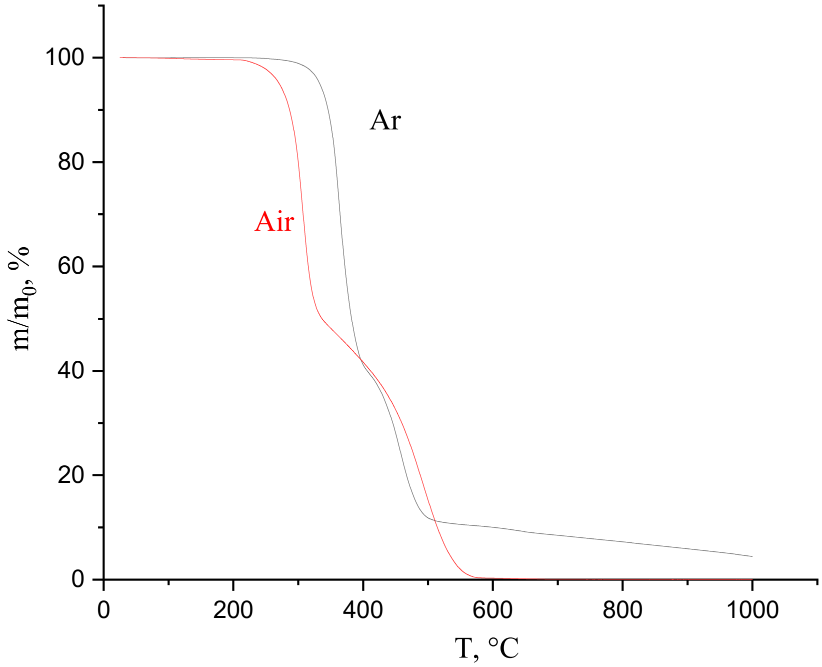


**Figure S15.** TGA curves for **PM2.**

## 5. DMA curves of the synthesized vinyl-addition homopolymers

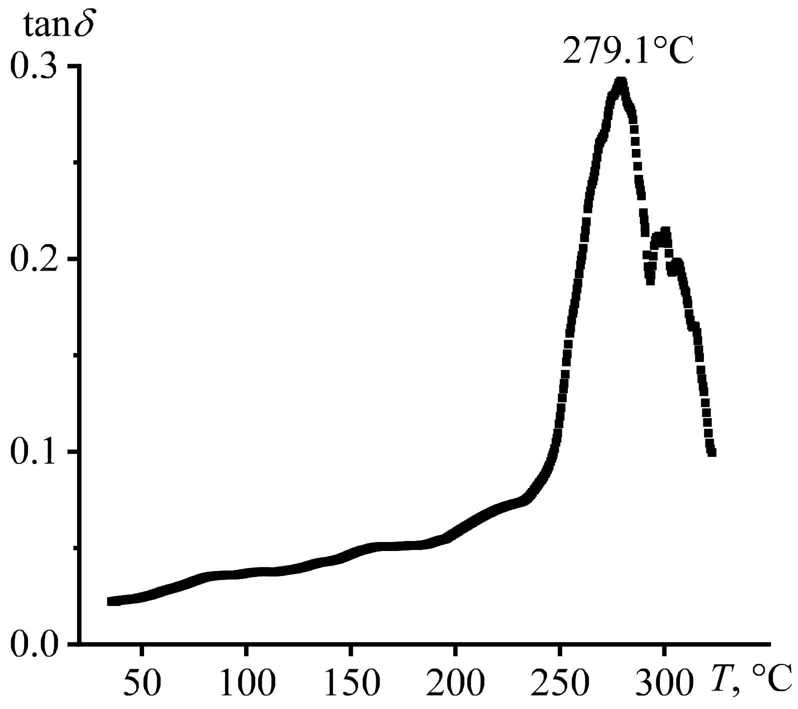


**Figure S16.** DMA curves of the synthesized **PM1**.

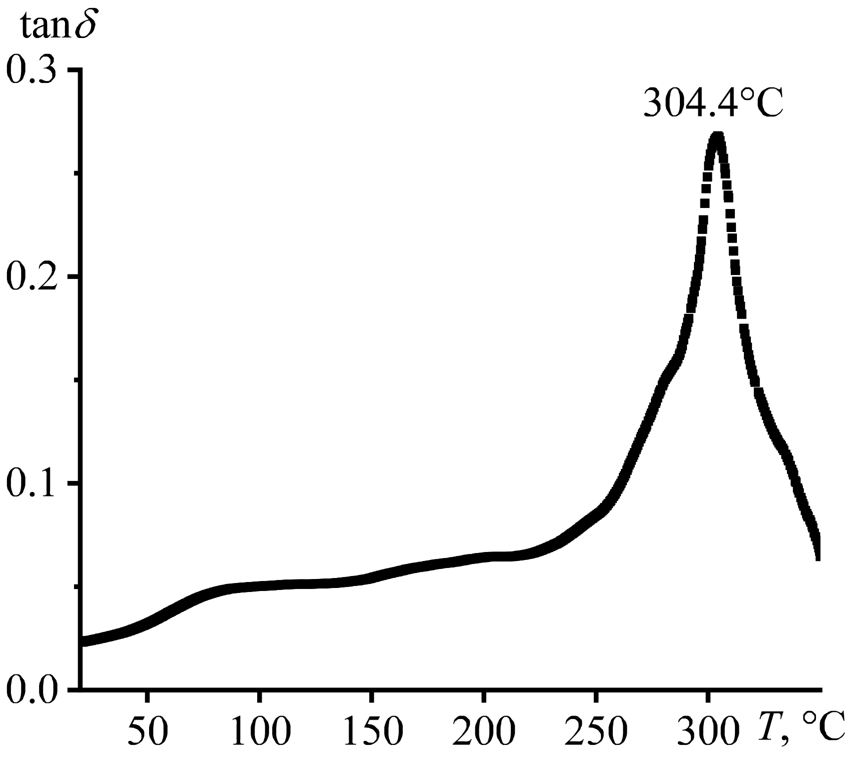


**Figure S17.** DMA curves of the synthesized **PM2**.

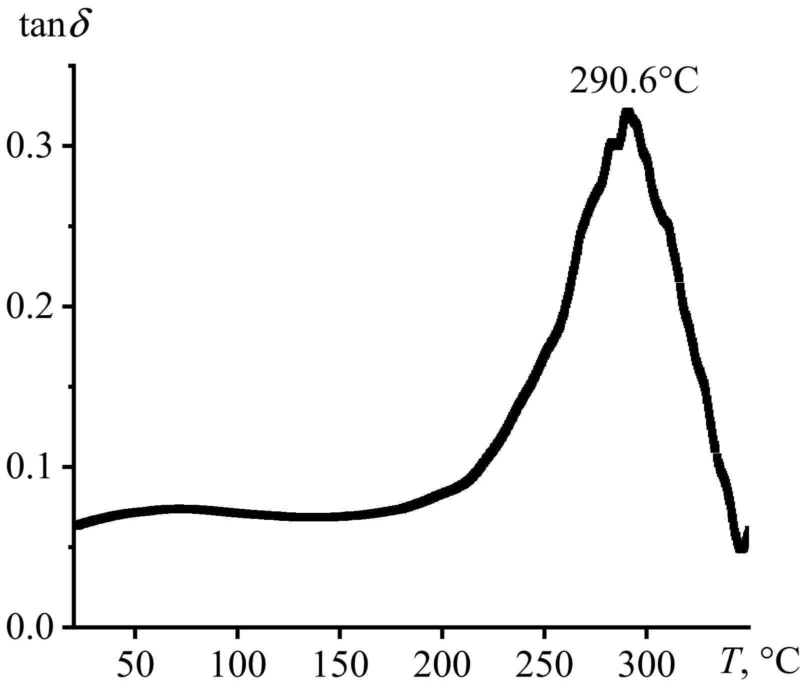


**Figure S18.** DMA curves of the synthesized **PM4**.

## References

1. Bermesheva, E.V.; Medentseva, E.I.; Khrychikova, A.P.; Wozniak, A.I.; Guseva, M.A.; Nazarov, I.V.; Morontsev, A.A.; Karpov, G.O.; Topchiy, M.A.; Asachenko, A.F.; Danshina, A.A.; Nelyubina, Y.V.; Bermeshev, M.V. Air-Stable Single-Component Pd-Catalysts for Vinyl-Addition Polymerization of Functionalized Norbornenes. *ACS Catalysis* **2022**, *12*, 15076-15090, doi:10.1021/acscatal.2c04345.
2. Marion, N.; Navarro, O.; Mei, J.; Stevens, E.D.; Scott, N.M.; Nolan, S.P. Modified (NHC)Pd(allyl)Cl (NHC = N-Heterocyclic Carbene) Complexes for Room-Temperature Suzuki−Miyaura and Buchwald−Hartwig Reactions. *J. Am. Chem. Soc.* **2006**, *128*, 4101-4111, doi:10.1021/ja057704z.
3. Viciu, M.S.; Zinn, F.K.; Stevens, E.D.; Nolan, S.P. Telomerization of Amines Mediated by Cationic N-Heterocyclic Carbene (NHC) Palladium Complexes. *Organometallics* **2003**, *22*, 3175-3177, doi:10.1021/om030337k.
4. Kolychev, E.L.; Asachenko, A.F.; Dzhevakov, P.B.; Bush, A.A.; Shuntikov, V.V.; Khrustalev, V.N.; Nechaev, M.S. Expanded ring diaminocarbene palladium complexes: synthesis, structure, and Suzuki–Miyaura cross-coupling of heteroaryl chlorides in water. *Dalton Transactions* **2013**, *42*, 6859-6866, doi:10.1039/c3dt32860k.
